# Açaí (*Euterpe oleracea* Mart.) Attenuates Oxidative Stress and Alveolar Bone Damage in Experimental Periodontitis in Rats

**DOI:** 10.3390/antiox11101902

**Published:** 2022-09-26

**Authors:** Vinicius Ruan Neves dos Santos, Deborah Ribeiro Frazão, Railson de Oliveira Ferreira, Paulo Fernando Santos Mendes, Daiane Claydes Baia-da-Silva, Deiweson Souza-Monteiro, Leonardo Oliveira Bittencourt, João Daniel Mendonça de Moura, José Messias Perdigão, Bruno José Brito Teixeira, Everton Luiz Pompeu Varela, Gabriela de Souza Balbinot, Sandro Percário, Herve Rogez, Cassiano Kuchenbecker Rösing, Fabrício Mezzomo Collares, Rafael Rodrigues Lima

**Affiliations:** 1 Laboratory of Functional and Structural Biology, Institute of Biological Sciences, Federal University of Pará, Belém 66075-110, PA, Brazil; 2 Center for Valorization of Amazonian Bioactive Compounds, College of Biotechnology, Federal University of Pará, Belém 66075-110, PA, Brazil; 3 Oxidative Stress Research Laboratory and Post-Graduate Program in Biodiversity and Biotecnology of the BIONORTE Network, Institute of Biological Sciences, Federal University of Pará, Belém 66075-110, PA, Brazil; 4 Dental Materials Laboratory, Department of Conservative Dentistry, School of Dentistry, Federal University of Rio Grande do Sul, Porto Alegre 90010-150, RS, Brazil; 5 Department of Periodontology, Faculty of Dentistry, Federal University of Rio Grande do Sul, Porto Alegre 90010-150, RS, Brazil

**Keywords:** periodontitis, antioxidants, açaí, *Euterpe oleracea*

## Abstract

Açaí (*Euterpe oleracea* Mart.) juice is rich in phenolic compounds with high antioxidant capacity. It has been observed that the use of antioxidants may be an additional strategy to nonsurgical periodontal therapy as well as to prevent alveolar bone loss. Thus, the objective of this study was to investigate the effects of açaí supplementation on experimental periodontitis in rats. Twenty male *Rattus norvegicus* (Wistar) rats were assigned into control, açaí, experimental periodontitis, and experimental periodontitis with açaí supplementation groups. Periodontitis was induced by placing ligatures around the lower first molars. Animals in the açaí groups received 0.01 mL/g of clarified açaí juice for 14 days by intragastric gavage. At the end of the experimental period, blood was collected to assess the reduced glutathione (GSH), Trolox equivalent antioxidant capacity (TEAC), and lipid peroxidation (TBARS) levels. Moreover, hemimandibles were analyzed by micro-computed tomography (micro-CT) for alveolar bone loss and bone quality. Açaí supplementation increased blood total antioxidant capacity and decreased lipid peroxidation. It also reduced alveolar bone loss when compared to the experimental periodontitis group. Moreover, clarified açaí per se modulated the oxidative biochemistry and bone microstructure. Thus, açaí may be considered a viable alternative for managing periodontal oxidative stress and preventing alveolar bone loss.

## 1. Introduction

**Figure 1:**
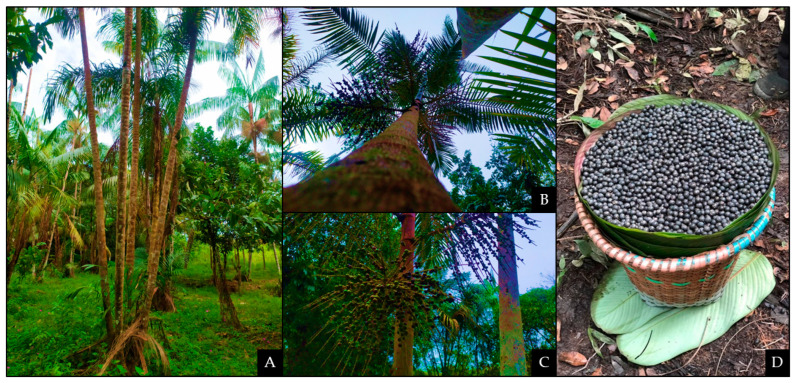
Açaí palm. In (**A**), The trunk of *Euterpe oleracea* Martius palm; In (**B**), view of the açaí palm canopy; In (**C**), açaí fruit in the ripening stage; In (**D**), black açaí fruit, the stage at which it is used to prepare the pulp.

*Euterpe oleracea* Martius palm tree (Figure 1A), commonly called açaí palm tree, is abundant along the Amazon basin soils, especially in its eastern part [1]. Açaí fruit is a globose sessile drupe with a violet/purple color when ripe, with a diameter of 1 to 2 cm, and an average weight of 0.8 to 2.3 g. The palm tree fruit, the açaí (Figure 1C,D), is widely consumed in pulp form by Brazil’s northern region population and has significant economic importance [2]. The preparation of açaí juice is a two-stage process: the fruit is softened in warm water, followed by mechanical pulping as water is added [3].

Phenolic compounds are the predominant phytochemicals in the açaí fruit [4]. The purple color of the fruit is due to the high concentration of anthocyanins, phenolic compounds belonging to the flavonoid class. When the fruit is fully ripened, the major polyphenols are the anthocyanins cyanidin-3-rutinoside and cyanidin-3-glycoside, followed by the non-anthocyanin compounds homoorientin, orientin, taxifolin deoxyhexose, vitexin, and isovitexin [5,6]. The composition and high concentration of phenolic compounds account for the high antioxidant capacity of the açaí fruit pulp, whether measured by the Oxygen Radical Absorbance Capacity (ORAC) or Total Oxidant Scavenging Capacity (TOSC), compared to other berries or any other fruit or vegetable [2]. In this context, açaí has already demonstrated several important systemic properties associated with the phenolic compounds, such as a role neural protection [7] and tissue repair [8], in addition to antioxidant defense [9], as an inhibitor of osteoclast activity [10], and in the reduction of oxidative stress [11,12].

Antioxidant agents have been studied as adjuvant therapies for the prevention and treatment of many diseases, such as periodontitis [13,14,15]. In periodontitis, the use of these agents has been associated with an improved endothelial function, a decrease in markers of inflammation, and an increase in the antioxidant capacity of the intrinsic glutathione system and overcoming of oxidative effects, thereby reducing treatment side effects and possibly minimizing periodontal breakdown [16]. Furthermore, it has been demonstrated that nutritional interventions in the management of systemic inflammatory diseases, such as fruit intake, vitamins, and fish oils, can enhance the body’s antioxidant and anti-inflammatory potential. Thus, there are already reports in the literature of the action of nutraceutical agents such as vitamin C, coenzyme Q10, and curcumin derived from turmeric as adjuvants for periodontal treatment [17,18]. In this regard, dietary intervention centered on antioxidants may minimize periodontal damage caused by an imbalance between oxidants and antioxidants, hence improving periodontitis clinical parameters [19].

Periodontitis is a multifactorial chronic inflammatory condition of supporting tissues of the teeth triggered by dysbiotic biofilms, and mediated by a host’s inflammatory response, and the susceptibility of the immunological system to environmental factors [20]. Periodontitis progression can result in changes in oxidative balance, bone metabolism with the disruption of osteoblast and osteoclast activity, collapse of the teeth-supporting apparatus and, ultimately, tooth loss [21]. Moreover, periodontitis is also associated with several systemic conditions such as diabetes mellitus, lupus, cancer, rheumatoid arthritis, respiratory, cardiovascular, and renal diseases through a chronic low-level systemic inflammation [22,23,24].

From this perspective, considering the already known antioxidant properties of açaí components under oxidative stress conditions and its promising protective effect, the objective of this study was to investigate the effect of açaí supplementation on the modulation of experimental periodontitis in rats, evaluating its ability to modulate prooxidant and antioxidant parameters associated with the development of periodontitis, and to manage periodontal breakdown. The present study successfully demonstrated that açaí supplementation prevented experimentally-induced alveolar bone loss.

## 2. Materials and Methods

### 2.1. Production and Composition of Clarified Açaí

The juice of *Euterpe oleracea* fruits used in this work was prepared according to a patented process (PI 1003060-3, 4 August 2010). Briefly, clarified açaí was prepared from fresh drupes. After cleaning the fruit, pulping was performed with the addition of 0.5 L of water per kilogram of fruit. The juice was subsequently microfiltered and clarified to obtain a thin, translucent, wine-colored liquid without lipids, proteins, or fibers but rich in phenolic compounds.

An aliquot of clarified açaí was characterized by total phenolics (TP) and anthocyanins composition. TP was determined by the Folin–Ciocalteu method [25]. Main flavonoid content was assessed using two validated UHPLC-DAD methods [5,6]. Orientin, homoorientin, taxifolin, vitexin, isovitexin, cyanidin 3-glucoside, and cyanidin 3-rutinoside (Extrasynthèse, Genay, France) were used as standard compounds.

### 2.2. Animals and Experimental Groups

This study was approved by the Ethics Committee on Animal Use of the Federal University of Pará (UFPA) (Report No. 2615120919). Twenty male *Rattus norvegicus* (Wistar) rats, 60 days old, weighing 150–200 g, obtained from the central animal house of UFPA were randomly assigned to four experimental groups (*n* = 5 per group): the control group, açaí group, experimental periodontitis (EP) group, and experimental periodontitis with açaí supplementation group (EP + açaí). Sample size estimation was based on Castro et al.’s (2020) study through G*Power software (Statistical Power Analyses 3.1.9.2). Animals were conditioned in a 12-h light/dark cycle, maintained at a controlled temperature (25 ± 1 °C), and received water from the same source and same food (NUVITAL^®^) ad libitum.

### 2.3. Induction of Experimental Periodontitis

To ensure similar stress conditions, on the first day of the experiment, all animals were submitted to intraperitoneal anesthesia with xylazine hydrochloride (8 mg/kg) and ketamine hydrochloride (75 mg/kg). After the loss of corneal reflexes of animals in the groups with experimental periodontitis, bandages with cotton ligatures were placed around the cervical regions of the first mandibular molars to induce periodontitis in groups exposed to experimental periodontitis, being maintained for 14 days until euthanasia [26,27,28].

### 2.4. Clarified Açaí Supplementation

Animals of the açaí groups received dosages of 0.01 mL per g of animal weight, after 24 h of cotton thread placement, for 14 days, by intragastric gavage [29]. Animals belonging to the other groups received a proportional volume of distilled water for the same period, also intragastrically.

### 2.5. Sample Collection

**Figure 2:**
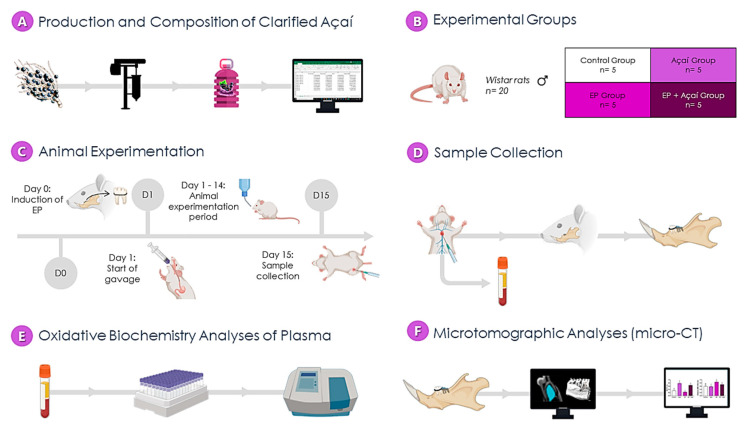
Flowchart of the experiment. (**A**)—the production stage and measurement of the composition of clarified açaí; (**B**)—allocation to experimental groups; (**C**)—experimental stages of the pre-collection study of the samples; (**D**)—sample collection stage, in which the plasm and the mandible were obtained; (**E**)—evaluation of plasm oxidative stress; (**F**)—microtomographic analysis (micro-CT).

At the end of the experimental period, the animals were anesthetized intraperitoneally with the same previously described protocol and had their blood collected by cardiac puncture with further centrifugation to plasma collection. Next, the animals were perfused with a heparinized (1%) saline solution (0.9%) and formaldehyde (4%) for fixation. The plasma was stored at −80 °C until further biochemical analyses and the left hemimandibles were post-fixed in 4% formaldehyde solution for microtomographic analyses. All methodological steps are summarized in Figure 2.

### 2.6. Biochemical Analysis

To evaluate açaí’s effects on blood oxidative biochemistry, blood samples were obtained before perfusing the animals, stored in tubes containing 50 µL of 5% ethylenediaminetetraacetic acid (EDTA), and centrifuged for 10 min at 3000 rpm. Plasma was collected and kept in Eppendorf tubes at −80 °C after centrifugation for subsequent investigation of reduced glutathione (GSH) levels, Trolox equivalent antioxidant capacity (TEAC), and thiobarbituric acid reactive substances (TBARS) levels, as previously described [30].

#### 2.6.1. Determination of Reduced Glutathione (GSH)

The determination of GSH concentrations was performed according to the method proposed by Ellman (1959) [31]. This method is based on the ability of glutathione present in the sample to reduce 5,5-dithiobis-2-nitrobenzoic acid (DTNB) to nitrobenzoic acid (TNB). The results were obtained as μg/mL and then converted to a percentage of control.

#### 2.6.2. Determination of Trolox Equivalent Total Antioxidant Capacity (TEAC)

TEAC value was determined following the RE et al., (1999) [32] method, the results expressed in μg/mL, and then converted to a percentage of control.

#### 2.6.3. Determination of Thiobarbituric Acid Reactive Substances (TBARS)

The samples were incubated with a thiobarbituric acid solution at 94 °C in a water bath for 60 min. After cooling at room temperature, n-butyl alcohol was added, then vortexed and centrifuged (2500 rpm, 10 min). The results were expressed in nM/mL and then converted to a percentage of control [33].

### 2.7. Micro-Computed Tomography (Micro-CT)

The left hemimandibles were subjected to micro-computed tomography (MicroCT.SMX-90 CT; Shimadzu Corp., Kyoto, Japan) to determine whether daily consumption of açaí can reduce periodontal breakdown. Thus, samples were placed on a rotating platform inside the device, and images were taken with 360° rotation at an intensity of 70 kV and 100 mA. Then, the images were reconstructed by inspeXio SMX-90CT software (Shimadzu Corp., Kyoto, Japan) with a voxel size of 10 µm in images at a resolution of 1024 × 1024 and 14 µm thickness, which resulted in 541 images per sample.

Bone images were taken in the interradicular region, close to the furcation region of the mandibular first molar. An area was standardized to create the region of interest (ROI), considering the interradicular region of the mandibular first molar from the apical third to the cervical third with an average area of 0.200 mm^2^. A threshold was applied to segment the different gray values present in the image. Furthermore, measurements were made with the software program ImageJ (National Institutes of Health, Bethesda, MD, USA). Differences in gray levels of bone and other structures in the images were considered to select the threshold. Based on this, the threshold was set from 120 to 255. Trabecular thickness (Tb.Th), trabecular separation (Tb.Sp), and bone volume to tissue volume ratio (BV/TV) were measured with the BoneJ plugin [34].

The software RadiAnt DICOM Viewer 5.0.1 (Medicant, Poznan, Poland) was used for the three-dimensional (3D) reconstruction of the left hemimandible. The 3D images of the samples were placed in a standard position, where the buccal and lingual surfaces of the teeth could be observed. The distance between the cementoenamel junction and the alveolar bone crest was defined as an evaluation parameter to measure the possible effects on bone loss. Thus, bone loss was detected by measuring the distance between the cementoenamel junction and the alveolar bone crest at six points on the mandibular first molar (i.e., mesialvestibular and vestibular-medial disto-vestibular, mesial-lingual, lingual-medial, disto-lingual) and averaging these points.

### 2.8. Statistical Analysis

To test the homocedasticity of the data, the Shapiro–Wilk statistical test was performed. Then, a one-way ANOVA with *Tukey’s* post hoc test was applied for comparison among groups, considering a statistical significance level of *p* < 0.05. GraphPad Prism 8.0.2 software (San Diego, CA, USA) was used for all analyses.

## 3. Results

### 3.1. Analysis of Clarified Açaí Juice Composition

An aliquot of clarified açaí was previously characterized with a total content in phenolic compounds of 3143.12 mg Eq. gallic acid/L. Using HPLC-DAD methods, the major phenolic compounds of clarified açaí were identified and quantified as cyanidin-3-glucoside (112.20 mg/L), cyanidin-3-rutinoside (543.30 mg/L), homoorientin (184.15 mg/L), orientin (144.81 mg/L), taxifolin deoxyhexose (13.06 mg/L), vitexin (10.57 mg/L), and isovitexin (10.18 mg/L).

### 3.2. Daily Consumption of Açaí Modulated Systemic Oxidative Biochemistry in Rats Plasma

**Figure 3:**
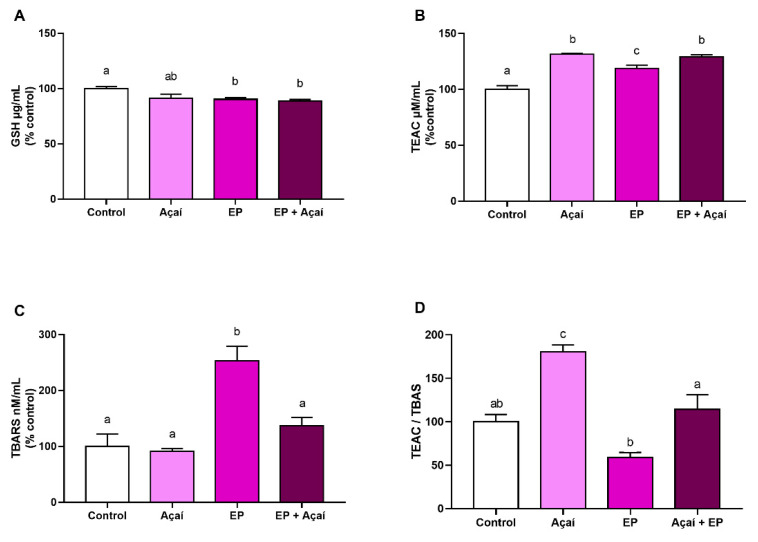
Oxidative biochemistry analyses. (**A**) Analyses of Reduced Glutathione (GSH); (**B**) Trolox equivalent antioxidant capacity (TEAC); (**C**) Thiobarbituric acid reactive substances (TBARS); (**D**) ratio between TBARS and TEAC. EP: Experimental Periodontitis. Results are expressed as a percentage (%) of control (mean ± S.E.M.). Different letters show a statistically significant difference (*p* < 0.05). One-way ANOVA test with Tukey’s post hoc test (*n* = 5).

The evaluation of plasma GSH levels showed that the experimental periodontitis group (90.18 ± 3.22%) presented lower levels of GSH in comparison to the control group (100 ± 3.49%; adj. *p*-value < 0.05). However, açaí supplementation did not demonstrate a statistically significant difference in comparison to the experimental periodontitis group (90.18 ± 3.22% vs. 88.66 ± 3.41%; adj. *p*-value = 0.71), as demonstrated in Figure 3A.

The experimental periodontitis with açaí supplementation group presented higher plasma TEAC levels than the experimental periodontitis group (129.23 ± 3.99% vs. 118.80 ± 2.71%; adj. *p*-value = 0.02). Interestingly, açaí per se (131.28 ± 0.83%) could also increase plasma TEAC levels compared to both control (100 ± 3.36%; adj. *p*-value < 0.0001) and experimental periodontitis groups (118.80 ± 2.71%; adj. *p*-value = 0.01; Figure 3B).

The group with experimental periodontitis (253.08 ± 51.78%) showed higher plasma TBARS levels in comparison to the control group (100 ± 43.72%; adj. *p*-value < 0.005) and, interestingly, the açaí supplementation in animals with experimental periodontitis (137.25 ± 31.87%) reduced the plasma TBARS levels in comparison to those animals not exposed to such supplementation (adj. *p*-value < 0.005; Figure 3C).

Regarding total antioxidant capacity and rate lipid peroxidation in plasma, the experimental periodontitis with açaí supplementation group demonstrated an increase in its values compared with the group with experimental periodontitis without supplementation (114.31 ± 16.88% vs. 59.0 ± 5.87%; adj. *p*-value = 0.002), suggesting a greater defense against oxidative damage. Moreover, the group without periodontitis, supplemented with açaí, had a rise in plasma TEAC/TBARS levels, compared to control (180.57 ± 7.89% vs. 100 ± 8.54%; adj. *p*-value = 0.005), experimental periodontitis (180.57 ± 7.89% vs. 59.0 ± 5.87%; adj *p*-value = < 0.0001), and experimental periodontitis with açaí supplementation group (180.57 ± 7.89% vs. 114.31 ± 16.88%; adj. *p*-value = 0.008; Figure 3D).

### 3.3. The Ingestion of Açaí Was Able to Reduce Periodontal Breakdown

The experimental periodontitis group presented the highest alveolar bone loss in the present study, demonstrated by the distance between the cementoenamel junction and alveolar bone crest (0.86 ± 0.04 mm). Supplementation with clarified açaí juice minimized that damage by reducing the alveolar bone loss (0.70 ± 0.01 mm) in comparison to the experimental periodontitis group (0.86 ± 0.04 mm; adj. *p*-value = 0.005). It is worth mentioning that the experimental periodontitis supplemented with açaí group did not statistically differ from the control group (0.70 ± 0.01 mm vs. 0.58 ± 0.02 mm; adj. *p*-value = 0.06).

Regarding bone quality parameters, when it comes to trabecular thickness (Tb.Th), the experimental periodontitis with açaí supplementation group demonstrated higher values compared to the experimental periodontitis group (0.15 ± 0.004 mm vs. 0.08 ± 0.006 mm; adj. *p*-value = 0.0002). Nevertheless, there was no statistically significant difference between the experimental periodontitis with açaí supplementation and control groups (0.15 ± 0.004 mm vs. 0.12 ± 0.01 mm; adj. *p*-value = 0.11).

**Figure 4:**
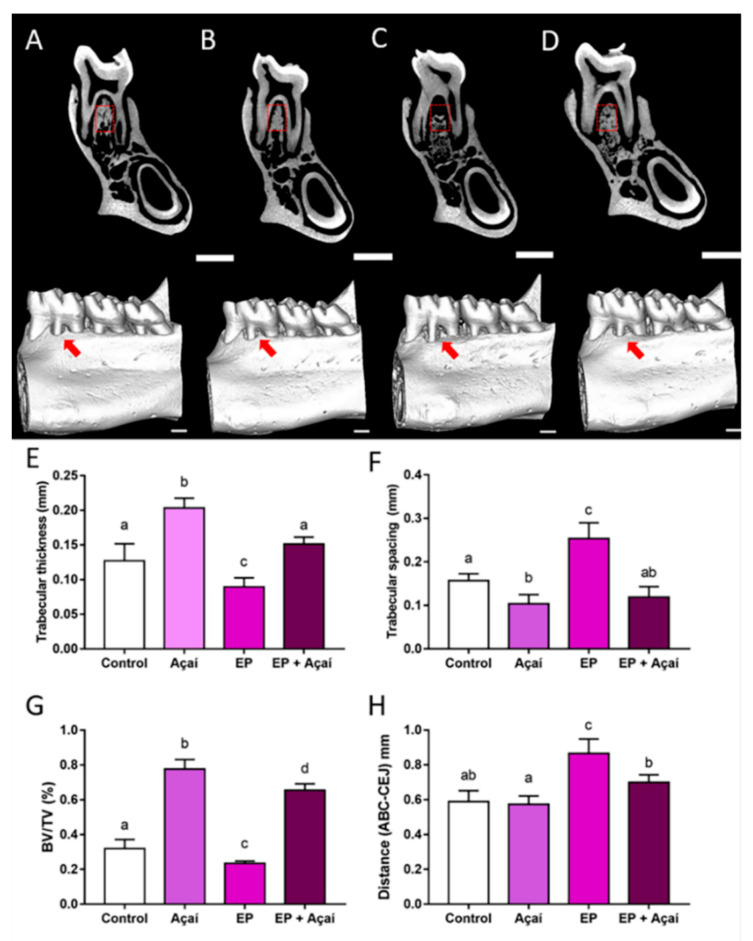
Effects of clarified açaí juice (0.01 mL/g/day for 14 days) on alveolar bone of rats (*n* = 5/group) exposed or not to experimental periodontitis. Sagittal slices of the animals hemimandibles with a red square representing the region of interest (ROI) and three-dimensional reconstructions of the hemimandibles with a red arrow highlighting the alveolar bone loss differences between the (**A**) control, (**B**) açaí, (**C**) experimental periodontitis, and (**D**) experimental periodontitis + açaí groups. Scale bar = 1 mm. (**E**) trabecular thickness (Tb.Th; mm); (**F**) trabecular spacing (Tb.Sp; mm); (**G**) bone volume to tissue volume (BV/TV; %); (**H**) alveolar bone crest to cementoenamel distance (ABC-CEJ; mm). The results are expressed as mean ± standard deviation. Different letters indicate a significant statistical difference. One-Way ANOVA followed by Tukey’s post hoc test, *p* < 0.05.

Moreover, the experimental periodontitis with açaí supplementation group demonstrated lower trabecular spacing (Tb.Sp) values compared to the experimental periodontitis group (0.11 ± 0.02 mm vs. 0.25 ± 0.036 mm; adj. *p*-value < 0.0001). The açaí group also had lower levels compared to controls (10.38 ± 0.01 mm vs. 0.15 ± 0.007 mm; adj. *p*-value = 0.03), as shown in Figure 4F.

The two groups supplemented with açaí showed higher levels of BV/TV (açaí group: 0.7772 ± 0.05409; experimental periodontitis + açaí group: 0.6552 ± 0.0368) in comparison to the groups without supplementation (control group: 0.3192 ± 0.05264; experimental periodontitis group: 0.2345 ± 0.01325; adj. *p*-value < 0.05; Figure 4G).

## 4. Discussion

This is a pioneering study in assessing the potential of clarified açaí juice in the management of periodontal breakdown by the reduction of bone loss and increasing bone quality, in an experimentally induced periodontitis model in rats. In addition to these findings, the systemic oxidative stress triggered by periodontitis was also attenuated by açaí supplementation. The clarified açaí juice modulated bone quality by increasing trabecular thickness and the bone volume to tissue volume ratio and decreasing trabecular spacing, suggesting that this natural product is a promising adjuvant for the prevention and treatment of periodontal disease. Furthermore, açaí supplementation was also capable of preventing additional alveolar bone loss, with levels similar to control.

Among the existing models of induced periodontitis, three are generally performed by authors: 1- ligature-induced periodontitis: insertion of a nylon, silk, or cotton thread in the cervical region of molars [35]; 2- lipopolysaccharide (LPS)-induced periodontitis—application of LPS in the marginal gingiva of molars [36] and; 3- microorganism-induced periodontitis—specific periodontal pathogens are applied to the marginal gingiva [37]. The inoculation of LPS and periodontopathogens is technically easier to perform due to difficulties in accessing the oral cavity of rodents. However, periodontitis results from the interaction between complex colonization of microorganisms, the host response, and several factors that can attenuate or worsen the loss of tissue attachment. Thus, the inoculation of LPS or bacteria represents a small part of the periodontal disease complexity. The ligature-induced periodontitis model implies a dental biofilm accumulation triggering an inflammatory response. *Actinomyces*, *Prevotella nigrescens*, *Fusobacterium*, *Porphyromonas gingivalis*, and *Aggregatibacter actinomycetemcomitans* constitute the biofilm that accumulates in the ligature [38]. Thus, the induction of experimental periodontitis in rats is a validated method due to its similarity to human periodontitis. In both cases, periodontitis damage in the destructive phase is marked by an inflammatory infiltrate in the gingiva, which occurs before bone resorption [38].

Micro-computed tomography (micro-CT) has become a widespread method to evaluate dentoalveolar structures in periodontal research. Since periodontitis causes an inflammatory disarrangement of periodontal tissues, especially a bone disruption, micro-CT is precise and currently considered the gold standard for assessing periodontal and bone microarchitecture changes [39]. Scanning parameters, such as a voltage of 70 KVp, voxel size of 6–10 μm, and integration time up to 300 ms, generate images with clear demarcation of bone resorption sites. The first mandibular molar is one of the most common teeth analyzed and reported as a reasonable model for a translational analysis of bone loss. Furthermore, according to a recent guideline for micro-CT analysis, the linear measurement of the distance from the cementoenamel junction to the alveolar bone crest (CEJ-ABC) is feasible in the assessment of bone loss generated by retained periodontal biofilm in the first molar [40].

In vitro studies showed that the extract of *Euterpe oleracea* causes a downregulation in NF-κB and its target genes, such as TNF-α, IL-6, IL-8, and IL-1β, which actively participate in the bone resorption processes; in addition, it inhibits the activity and differentiation of osteoclasts, acting on the activity of RANK-L cells, modulating inflammatory cytokines, decreasing the secretion of IL-1α, IL-6, and TNF-α, and increasing the secretion of IL-3, IL-4, IL-14, and IFNγ [10,41]. Systemically, açaí supplementation has also been demonstrated to influence essential parameters in the bone resorption process during the formation of the periradicular lesion, such as the reduction of TNF-α expression [42,43]; IL-1β and IL-6 [43]); IL 8 and NF-κB (14); prevention of oxidative damage by a direct mechanism [44]; and positive impact on serum antioxidant enzyme activity [9]. Although we did not assess the inflammatory condition, our findings suggest that açaí can modify bone injury by influencing the inflammatory response via oxidative stress modulation.

A benefit of *Euterpe oleracea* is the ability to modulate the expression of the inducible nitric synthase enzyme (iNOS), reducing the inflammatory response in macrophage cell cultures [45]. In addition to free radical scavenging action (radical peroxyl, peroxynitrite, and hydroxyl radical), the mechanism involved in this action is associated with the presence of anthocyanins and other flavonoids [46,47]. Furthermore, flavonoids are related to increases in the production and activity of antioxidant enzymes such as SOD, GPx, and catalase, which help reduce ROS [48]. ROS are produced by different inflammatory cells in periodontitis [49], which perform the host response to microbial aggression. The primary sources of ROS are PMNs and fibroblasts, both contributing to the establishment and progression of periodontal tissue destruction [50].

Many studies using the ligature-induced periodontitis model have found malonaldehyde as a marker of lipid peroxidation in serum, plasma, and tissue homogenates [49,51,52,53,54]. When superoxide dismutase is increased, ligature-induced inflammation and the increase in malondialdehyde can be considerably decreased [55,56]. The plasma total antioxidant activity and glutathione had higher levels in the periodontitis with açaí supplementation group in our study. As SOD can increase glutathione levels [49,57], we can extend the hypothesis that açaí is linked to a reduction in lipid peroxidation levels by increasing the total antioxidant activity associated with more significant sequestration of reactive oxygen species.

Oxidative stress is intrinsically related to periodontal connective tissue damage. Cytokines, matrix metalloproteinase activity, and superoxide radicals are observed in the loss of periodontal tissue attachment, and are also increased in bone resorption [49,51]. Periodontal treatment reduces superoxide levels in periodontal tissues leading to reductions in damage. Therefore, additional ways to reduce oxidative stress may positively impact bone preservation periodontal therapy [50,57]. These findings also support the role of açaí in the prevention of periodontal breakdown. The alveolar bone is the most dynamic tissue in the periodontium, and its structural characteristics are directly related to its functions and health [58,59]. Changes in the alveolar bone microarchitecture are observed in rats with induced periodontitis, with consequences on bone quality [26,27]. Our results are in accordance with that, as the group with experimental periodontitis showed alterations in trabecular thickness, spacing, and bone volume to tissue volume ratio compared to the control group (Figure 4). However, rats with the açaí supplementation improved bone quality parameters by increasing trabecular thickness and decreasing the trabecular spacing associated with the rise in the BV/TV parameter, compared to the groups exposed or not to experimental periodontitis. The alveolar bone commonly presents a highly mineralized bone mass with a trabecular architecture, showing thick bone with reduced space [58]. Hence, our data suggest that açaí may play a role in the modulation of alveolar bone maturity and could attenuate the damage caused by the periodontitis model in this study.

Furthermore, bone loss may be a response related to the morphological bone state and the activity of bone cells in the course of the inflammatory process of periodontitis [59]. Our data showed that açaí supplementation decreased alveolar bone loss observed in rats exposed to experimental periodontitis. This could be associated with the bone microstructure pattern found in the animals and the biochemical assay results since polyphenols found in açaí can act in osteoclastogenesis pathways [60].

The nuclear factor kappa B NF-κB pathway has been used to identify a reduction in osteoclast activity by inhibiting hypoxia-inducible factor-1 (HIF-1a) expression in an in vitro model [60]. Hypoxia in periodontitis is caused by the destruction of periodontal tissues, including vascular tissue injury, decreased local blood flow, and edema caused by the inflammatory process [20,52]. Hypoxic cells release HIF-1, which is regulated by the NF-κB pathway, and osteoclast activation occurs, primarily because of an increase in RANKL expression and a decrease in OPG in hypoxic cells.

Our findings raise additional questions about the açaí supplementation’s translational applicability in humans. Furthermore, we wonder if its effects extend beyond the oxidative biochemical system to the inflammatory response and possibly immune modulation in response to pathogenic microbiota. Finally, further clinical trials are needed to verify its translational applicability in humans.

## 5. Conclusions

Açaí supplementation protects against oxidative damage by reducing the formation of lipid peroxidation products, thus suggesting a potential protective effect promoted by the antioxidants present in açaí. Moreover, daily supplementation of clarified açaí resulted in a significant reduction in alveolar bone loss and changes in trabecular thickness, bone volume to tissue volume ratio, and trabecular spacing. Therefore, açaí antioxidant effects make it a potential adjuvant for the prevention and, eventually, treatment of periodontitis. This study raises new questions regarding the therapeutical potential of açaí.

## Data Availability

All data are available in the article.
